# Supplementary material for: A role for P-selectin and complement in the pathological sequelae of germinal matrix hemorrhage
Source: J Neuroinflammation. 2023 Jun 16;20:143. doi: 10.1186/s12974-023-02828-4 (PMC10273747; doi:10.1186/s12974-023-02828-4)
Supplement: Supplementary file 5 — Additional file 5. Video captions. [file 12974_2023_2828_MOESM5_ESM.docx]

**Additional file 1.**

Video showing representative microglia from vehicle treated GMH mice, reconstructed in 3D space with IF masking and modeling shown in Figure 4b.

**Additional file 2.**

Video showing representative microglia from 2.3Psel-Crry treated GMH mice, reconstructed in 3D space with IF masking and modeling shown in Figure 4c.

**Additional file 3.**

Video showing representative microglia from vehicle treated GMH mice, reconstructed in 3D space with IF masking and modeling shown in Figure 4d (left).

**Additional file 4.**

Video showing representative microglia from Psel-Crry treated GMH mice, reconstructed in 3D space with IF masking and modeling shown in Figure 4d (right).
